# Reproducibility of mRNA-Based Testing of *ESR1*, *PGR*, *ERBB2*, and *MKI67* Expression in Invasive Breast Cancer—A Europe-Wide External Quality Assessment

**DOI:** 10.3390/cancers13184718

**Published:** 2021-09-21

**Authors:** Ramona Erber, Arndt Hartmann, Peter Andreas Fasching, Matthias Ruebner, Robert Stöhr, Matthias Wilhelm Beckmann, Miriam Zentgraf, Verena Popp, Jodi Weidler, Iris Simon, Steffi Becker, Hanna Huebner, Josephine Fischer, Elena Guerini Rocco, Giuseppe Viale, Anne Cayre, Frederique Penault-Llorca, Tamara Caniego Casas, Belén Pérez-Miés, José Palacios, Paul Jank, Carsten Denkert, Lina Khoury, Thomas Mairinger, Fulvia Ferrazzi

**Affiliations:** 1 Institute of Pathology, University Hospital Erlangen, Friedrich-Alexander-Universität Erlangen-Nürnberg (FAU), Comprehensive Cancer Center Erlangen-EMN (CCC ER-EMN), 91054 Erlangen, Germany; robert.stoehr@uk-erlangen.de (R.S.); miriam.zentgraf@gmx.net (M.Z.); verena.popp@uk-erlangen.de (V.P.); fulvia.ferrazzi@uk-erlangen.de (F.F.); 2 Department of Gynecology and Obstetrics, University Hospital Erlangen, Comprehensive Cancer Center Erlangen-EMN (CCC ER-EMN), Friedrich-Alexander-Universität Erlangen-Nürnberg (FAU), 91054 Erlangen, Germany; peter.fasching@uk-erlangen.de (P.A.F.); matthias.ruebner@uk-erlangen.de (M.R.); matthias.beckmann@uk-erlangen.de (M.W.B.); hanna.huebner@uk-erlangen.de (H.H.); 3 Cepheid, Department of Medical and Scientific Affairs and Strategy, Oncology, 904 Caribbean Drive, Sunnyvale, CA 94089, USA; Jodi.Weidler@cepheid.com (J.W.); irissimon@yahoo.com (I.S.); Steffi.Becker@cepheid.com (S.B.); 4 Qualitätssicherungs-Initiative Pathologie QuIP GmbH, 10117 Berlin, Germany; Fischer@quip.eu; 5 Department of Pathology and Laboratory Medicine, IEO, European Institute of Oncology, IRCCS, 20141 Milan, Italy; Elena.GueriniRocco@ieo.it (E.G.R.); giuseppe.viale@ieo.it (G.V.); 6 Department of Oncology and Hemato-Oncology, University of Milan, 20122 Milan, Italy; 7 Centre Jean Perrin, Department of Pathology and Clermont Auvergne University, INSERM, U1240, “Imagerie Moléculaire et Stratégies Théranostiques”, F-63011 Clermont Ferrand, France; Anne.CAYRE@clermont.unicancer.fr (A.C.); Frederique.PENAULT-LLORCA@clermont.unicancer.fr (F.P.-L.); 8 Instituto Ramón y Cajal de Investigación Sanitaria, 28034 Madrid, Spain; tamara880723@hotmail.com (T.C.C.); bperezmies@gmail.com (B.P.-M.); jose.palacios@salud.madrid.org (J.P.); 9 CIBER-ONC, Instituto de Salud Carlos III, 28029 Madrid, Spain; 10 Department of Pathology, Hospital Universitario Ramón y Cajal, 28034 Madrid, Spain; 11 Facultad de Medicina, Universidad de Alcalá de Henares, 28871 Madrid, Spain; 12 Institute of Pathology, UKGM—University Hospital Marburg, Philipps-University Marburg, 35043 Marburg, Germany; paul.jank@uni-marburg.de (P.J.); carsten.denkert@uni-marburg.de (C.D.); 13 MVZ Helios Hospital Emil von Behring GmbH, 14165 Berlin, Germany; lina.khoury@helios-gesundheit.de (L.K.); thomas.mairinger@helios-gesundheit.de (T.M.); 14 Department of Nephropathology, Institute of Pathology, Friedrich-Alexander-Universität Erlangen-Nürnberg (FAU), 91054 Erlangen, Germany

**Keywords:** breast cancer, *ESR1*, *PGR*, *ERBB2*, *MKI67*, mRNA expression testing, STRAT4, GeneXpert, Xpert

## Abstract

**Simple Summary:**

Four biomarkers [estrogen receptor (ER), progesterone receptor (PgR), Ki-67, and HER2], are used to stratify breast cancer (BC) into subtypes predictive of therapy response. In a Europe-wide external quality assessment, we compared performance of an mRNA-based method [Xpert^®^ Breast Cancer STRAT4 (CE-IVD)] for determining *ESR1*, *PGR*, *ERBB2*, and *MKI67* expression against the gold standard [immunohistochemistry (IHC)/HER2 in situ hybridization (ISH)]. The coordinating center (CC) and five European laboratories tested ten breast cancer samples. STRAT4 binary (positive or negative) results of each marker were compared with the gold standard. *ESR1* and *ERBB2* mRNA results were concordant with IHC/ISH in all single analyses. In contrast, *PGR* and *MKI67* results were discordant in a few cases, which had STRAT4 expression values close to assay cut-offs and immunohistochemically presented heterogeneous low positive PgR and heterogeneous Ki-67. STRAT4 assay may be a reproducible method. However, cases with expression values close to cut-offs should be carefully reviewed.

**Abstract:**

Estrogen receptor (ER), progesterone receptor (PgR), Ki-67, and HER2 immunohistochemistry (IHC) together with *HER2* in situ hybridization (ISH) are utilized to classify invasive breast cancer (IBC) into predictive molecular subtypes. As IHC evaluation may be hampered by analytical errors, gene expression assays could offer a reliable alternative. In this first Europe-wide external quality assessment (EQA) study, we investigated performance of mRNA-based Xpert^®^ Breast Cancer STRAT4 (CE-IVD) in five European laboratories. The cohort comprised ten pre-therapy IBC core biopsies diagnosed in the coordinating center (CC). STRAT4 binary (positive or negative) mRNA results of each marker (*ESR1*, *PGR*, *ERBB2,* *MKI67*) were compared with the gold standard IHC/ISH performed by the CC. Sensitivity, specificity, and accuracy of *ESR1* and *ERBB2* mRNA were 100% for all samples. In contrast, *PGR* expression was falsely negative for one case by two sites and *MKI67* falsely negative for two cases (respectively by four and one sites). These cases had STRAT4 expression values close to assay cut-offs and immunohistochemically presented heterogeneous low positive PgR and heterogeneous Ki-67. Our EQA shows that STRAT4 mRNA assay may be a reproducible method to evaluate ER, PgR, HER2, and Ki-67 status. However, cases with expression values close to assay cut-offs should be carefully reviewed.

## 1. Introduction

Invasive breast cancer (IBC) can be classified into different gene expression-based subtypes, namely Luminal A, Luminal B, HER2-enriched, and basal-like, which are associated with different prognosis and different response to therapy [1,2,3]. In clinical routine, the immunohistochemical (IHC) determination of estrogen receptor (ER, gene/mRNA *ESR1*), progesterone receptor (PgR, gene/mRNA *PGR*), human epidermal growth factor receptor 2 (HER2, gene synonym: *ERBB2*) and Ki-67 (gene/mRNA *MKI67*) are used to approximate these molecular subtypes. Using these markers, IBC can be classified into molecular-like breast cancer subtypes: hormone receptor-positive tumors, which include Luminal A-like and Luminal B-like (either HER2-negative or HER2-positive), HER2-positive tumors (HER2+, non-luminal), and triple-negative breast cancer (TNBC) [4,5].

The IHC assessment of ER, PgR, HER2, and Ki-67 requires standardized staining and evaluation procedures. However, IHC protocols for each of these four biomarkers are linked to some difficulties: IHC staining may be hampered by many pre-analytical and analytical errors, e.g., inadequate fixatives/fixation, decalcification, age of archived unstained slides, sensitivity of antibody clones, and detection kits. In addition, discussions on the optimal IHC cut-offs for biomarker positivity are still on-going. Indeed, cut-offs that are clinically of prognostic and/or predictive value have to be assessed performing comprehensive clinical trials [6,7]. For ER/PgR, HER2, and Ki-67, cut-off recommendations have changed several times in the last 20 years [5,6,8,9,10,11,12,13,14,15,16,17,18,19]. Moreover, in the case of ER IHC, there is controversy on the current cut-off of ≥1%, since some studies have shown that IBCs with low ER expression (1–9% or 1–10%; cut-offs depending on different guidelines/references) behave more like ER-negative tumors [9,20,21,22]. Difficulties also exist for HER2, as in cases of HER2 IHC score equal to 2+ (borderline/equivocal), IHC has to be complemented by in situ hybridization (ISH), which is another very sensitive assay that requires standardized reading and optimal cut-offs [12]. Regarding Ki-67, a standardized protocol and cut-off are still lacking [6,15], although efforts to harmonize Ki-67 assessment have been published recently [23]. 

Due to the aforementioned issues, the pathological assessment of molecular-like breast cancer subtypes using IHC/ISH might be challenging, especially in low- and middle-income countries (LMIC). The use of a standardized, robust, reliable, and cost-effective mRNA-based test for the four biomarkers, ER (*ESR1*), PgR (*PGR*), HER2 (*ERBB2*), and Ki-67 (*MKI67*), may offer a good alternative to IHC. Some assays have already been validated and are commercially available [24,25]. Among these, the validity, robustness, and reproducibility of the Xpert^®^ Breast Cancer STRAT4 test (CE-IVD, in vitro diagnostic medical device. Not available in the U.S. Not available in all countries.) (“STRAT4”) (Cepheid, Sunnyvale, CA, USA) have been shown in large breast cancer cohorts [25,26,27]. Indeed, one blinded, retrospective study reported high concordance between biomarker expression detection by STRAT4 and IHC with receiver operating characteristic curve (ROC) area under the curve (AUC) values of 0.99, 0.95, 0.99, and 0.85 for *ESR1*, *PGR*, *ERBB2*, and *MKI67*, respectively [26]. However, in most European countries, regular participation in Europe-wide external quality assessment (EQA) programs is required for pathology labs and tumor centers to receive laboratory accreditation. Until now, there have been EQA studies for ER/PgR/HER2/Ki-67 by IHC and HER2 ISH testing, but not for mRNA-based assessment of *ESR1*/*PGR*/*ERBB2*/*MKI67*. Here, we present the first Europe-wide EQA study for one cartridge-based “four-marker” gene expression assay, the STRAT4 assay, evaluating its reproducibility and correlation to the gold standard (IHC/ISH) in different pathologic laboratories across Europe. 

## 2. Materials and Methods

### 2.1. Sample Cohort

Ten cases of diagnostic pre-therapy breast core biopsies with invasive breast cancer (IBC) that had been diagnosed in the coordinating center (CC) between 2016 and 2018 were included in the EQA study. These cases contained six ER+/PgR+ IBC with varying proliferation rates, (Ki-67), two HER2+, and two TNBC (Table 1). Each sample had to feature sufficient formalin-fixed and paraffin-embedded tissue to guarantee that sufficient diagnostic material would be left after the EQA study in case of necessary re-evaluation after tumor progression. Tumor cellularity had to be ≥30%. Moreover, cohort cases were not allowed to be included in any clinical trial. Pathological features, including histological tumor type, as well as IHC and ISH data for ER, PgR, HER2, and Ki-67 were extracted from the original pathology report. For each case, Xpert^®^ Breast Cancer STRAT4 (*ESR1*, *PGR*, *ERBB2,* and *MKI67*) mRNA test was performed in the CC and STRAT4 results for each marker had to match IHC/ISH subtyping. The Ethics Committee of the Medical Faculty of Erlangen University Hospital approved the study [ref. numbers 2700 (approval date: 1 March 2013) and 297_17 Bc (approval date: 11 January 2018)], and all patients provided written informed consent.

### 2.2. Immunohistochemical (IHC) Expression of ER, PgR, HER2, and Ki-67 and IHC Subtyping

IHC was conducted on FFPE IBC tissue of the preoperative core biopsies according to the established and accredited routine standards of the CC and manufacturer’s instruction manual with an automated staining module (BenchMark ULTRA IHC/ISH staining module, Ventana Medical Systems, Oro Valley, AZ, USA). For assessment of ER, PgR, and Ki-67 IHC status, a monoclonal rabbit antibody against ER-alpha (clone EP1, 1:40 dilution, DAKO, Glostrup, Denmark), a monoclonal mouse antibody against PgR (clone pgR636, 1:200 dilution, DAKO, Glostrup, Denmark), and a monoclonal antibody against Ki-67 (clone MIB-1, 1:100 dilution, DAKO, Glostrup, Denmark) were used. The continuous percentage of positively stained tumor cells was stated in the pathology reports. Moreover, staining intensity and the immunoreactive score (IRS) according to Remmele and Stegner were reported for ER and PgR [28]. For HER2 IHC staining, a polyclonal antibody against HER2 (1:200 dilution, DAKO, Glostrup, Denmark) was used, and HER2 IHC score was documented in the pathology reports as 0, 1+, 2+, or 3+ in accordance with the published recommendations [11]. Tumors with a score of 0 or 1+ were regarded as HER2-negative, and cases with a score of 3+ were considered as HER2 positive. Breast cancer samples with a 2+ staining were analyzed for gene copy number (GCN) alteration of *HER2* using chromogenic in situ hybridization (CISH). The *HER2* GCN and the centromere of the corresponding chromosome 17 were visualized using a kit with two probes of different colors (ZytoDot^®^ 2C SPEC *ERBB2*/*CEN17* Probe, ZytoVision GmbH, Bremerhaven, Germany). A case was defined as *HER2* amplified/positive if the *HER2*/*CEN17* ratio was ≥ 2.0 or if the *HER2* GCN was ≥ 6.0 signals per tumor nuclei [11]. 

IHC (and for HER2, IHC/CISH) binary (positive/negative) status was defined as follows:

- Samples with ER or PgR IHC ≥1% positive stained cells, regardless of staining intensity, were classified as positive (<10% as low positive); samples with <1% cells staining at any intensity were classified as negative [8,9,20,21]. ER and/or PgR positivity were summarized as hormone receptor positivity (HR+).
- Samples with HER2 IHC 0 or 1+ were classified as negative, HER2 IHC 3+ were defined as positive; cases with IHC 2+ results required reflex testing by CISH to determine positive or negative results [11,12,29].
- Samples with Ki-67 > 20% cells staining positive, regardless of staining intensity, were classified as high/positive. Samples with ≤ 20% cells staining at any intensity were counted as low/negative [5,14,30].

IHC/ISH breast cancer subtyping was concluded as follows: Cases that were negative for ER, PgR, and HER2 were regarded as TNBC, those with HER2 positivity as HER2+ subgroup, and IBC with ER and/or PgR positivity but HER2 negativity as HR+ subtype. This group was further divided into HR+ with low versus high Ki-67 expression. IHC status of each marker (ER, PgR, HER2, and Ki-67) and IHC/ISH subtyping of the ten EQA IBC samples are reported in Table 1.

### 2.3. mRNA-Based Testing of ESR1, PGR, ERBB2, and MKI67 in the Coordinating Center

The Xpert^®^ Breast Cancer STRAT4 (CE-IVD*) mRNA assay (Cepheid, Sunnyvale, CA, USA) is a Real Time-quantitative Polymerase Chain Reaction (RT-qPCR) based semi-quantitative assay to analyze *ESR1*, *PGR*, *ERBB2,* and *MKI67* mRNA levels isolated from FFPE IBC tissue. CC performed the test according to the manufacturer’s instructions. Archived FFPE blocks of the ten cases were used as starting material. For each block, one hematoxylin and eosin (H&E) stained slide containing a single 2 µm stained section for confirmation of breast cancer diagnosis and tumor area as well as one unstained slide containing a single 10 µm tissue section intended for mRNA testing were created. After reviewing the IBC and marking the tumor area on the H&E slide by a pathologist experienced in breast cancer, the FFPE tumor area was removed (scraped using a single use razor blade) from the unstained slide and then was prepared into a tissue lysate using the FFPE lysis kit (Cepheid, Sunnyvale, CA, USA) following the manufacturer’s instructions per the STRAT4 product insert. A 520 µL aliquot of the prepared tissue specimen lysate was then transferred to the sample chamber of a STRAT4 cartridge and put into a GeneXpert^®^ Instrument module for automated RNA extraction, purification, and RT-qPCR analysis [Cepheid, Sunnyvale, CA, USA, (http://www.cepheid.com/us/cepheid-solutions/systems/genexpert-systems/genexpert-i, accessed on 29 March 2020)]. The detailed protocol as well as the GeneXpert^®^ DX software analysis settings of the automated diagnostic platform have been previously described [26]. The assay uses cytoplasmic FMR1-interacting protein 1 (*CYFIP1)* as the sample adequacy control and reference gene to normalize the mRNA expression levels. In addition to the binary results and cycle threshold (Ct) values of the internal reference target *CYFIP1*, the software outputs delta cycle threshold (dCt) values, as well as binary test results, of each biomarker of interest (*ESR1*, *PGR*, *ERBB2,* and *MKI67*). The dCt value is defined as difference between the *CYFIP1* Ct and the Ct of the target gene (*C*t*_CYFIP_*_1_)−(*C*t_target gene_) [26]. The dCt value is then compared to a prespecified validated cut-off to define the binary (positive/negative) marker result for each target analyte.

### 2.4. EQA Implementation

Five European (France, Germany, Italy, Spain) pathology laboratories (here de-identified as EQA sites A-E) participated in the EQA study. All participants had already established the diagnostic platform and corresponding GeneXpert Dx software (including assay definition files) for STRAT4 testing in their laboratories and participated in a training for the Xpert^®^ Breast Cancer STRAT4 mRNA analysis (as was the case for the CC). Each site received one 2 µm thick H&E stained slide and one 10 µm thick adjacent unstained slide, prepared and shipped by the CC, for each of the 10 patient de-identified clinical test specimens for STRAT4 analysis. Xpert^®^ Breast Cancer STRAT4 kits and FFPE lysis reagents were directly provided by Cepheid. Each laboratory performed the mRNA assay according to the aforementioned criteria/protocol. Raw data of the test result were then transferred by participating laboratories directly to the CC for statistical analysis.

### 2.5. Re-Evaluation of Discrepant Cases

After the EQA analysis, for the cases that received discrepant results by the participating sites with respect to the CC, the CC H&E slide, as well as initial CC IHC stainings of the discrepant marker(s), were reviewed. Moreover, deeper sections (following the sections created for STRAT4 analysis at the five EQA sites) were cut, and IHC as well as STRAT4 analysis were repeated and compared with the initial CC results.

### 2.6. Statistical Analysis

Test results received from the participating centers were collected by the CC. In addition to the pathological parameters (tumor grading, IHC) of each EQA sample, binary mRNA test results as well as dCt values of the internal reference gene and each biomarker of interest (*ESR1*, *PGR*, *ERBB2* and *MKi67*), reported from each site, were recorded. 

Data analysis was performed in the R statistical environment v. 3.6.1 (R Core Team, 2019) [31]. Sensitivity, specificity, precision (positive predictive value), and accuracy for each marker and each of the five participating centers were calculated by comparing the binary test results with the IHC status assessed by the CC, taken as the gold standard. Confidence intervals for the average performance indicators across the five centers were calculated relying on a t distribution.

## 3. Results

### 3.1. Comparison of Binary STRAT4 Results

The primary objective of our study was to compare the binary results of each marker, separately for each center, with the binary (positive/negative) IHC status (and additionally HER2 CISH, if necessary) assessed by the CC, considered as the gold standard. Each site passed the technical quality criteria of the assay and received evaluable results from STRAT4 testing for each of the ten EQA samples and each marker. Overall, all centers passed the EQA study criteria, which required an agreement of at least 80% between test results for each marker and the CC IHC. The average sensitivity, specificity, and accuracy (overall concordance) of *ESR1* and *ERBB2* mRNA testing across all five participating sites were 100% for all samples. Instead, *PGR* mRNA had a discordant negative result for one case (case #2) by two sites, and thus had an average sensitivity of 94%, specificity of 100%, and accuracy of 96%. *MKI67* mRNA was discordant negative for two cases (case #2 by four sites, #10 by one site), thus having average sensitivity of 86%, specificity of 100% and accuracy of 90% (Table 2). 

**Figure 1:**
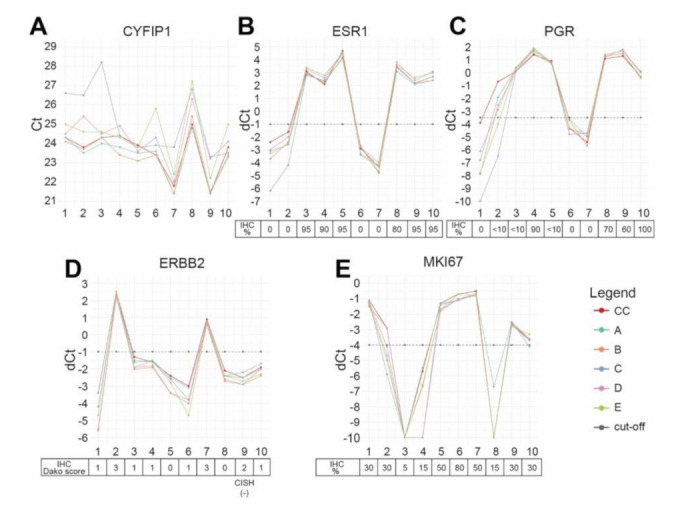
STRAT4 semi-quantitative mRNA level measurements for the reference gene and the four markers of interest. (**A**) Cytoplasmic FMR1-interacting protein 1 (*CYFIP1*) cycle threshold (Ct) measurements across the coordination center (CC) and the five participating sites A–E. (**B**–**E**) Delta cycle threshold (dCt) measurements for the markers across CC and the five participating sites A–E: (**B**) estrogen receptor (*ESR1*); (**C**) progesterone receptor (*PGR*); (**D**) human epidermal growth factor receptor (*ERBB2*); (**E**) Ki-67 (*MKI67*). Cut-off: dCt cut-off utilized by the STRAT4 assay.

The second study end point was to compare the quantitative Xpert^®^ Breast Cancer STRAT4 dCt results for each marker with the percentage staining results by IHC for ER, PgR and Ki-67 as well as HER2 categorical results (negative = IHC 0, 1+, 2+/ISH non-amplified versus positive = IHC 3+ or IHC 2+/CISH amplified). However, these analyses were for descriptive purposes and not required for passing the study. Across the coordination center and the EQA sites A–E, the reference gene *CYFIP1* cycle threshold (Ct) measurements were between 21–28 for all samples, which was well below the valid Ct cut-off of 35 (Figure 1A). Regarding *ESR1* and *ERBB2*, dCt measurements across all sites for all samples were comparable (Figure 1B,D). Instead, *PGR* dCt values for the IHC-positive case #2 were below the STRAT4 dCt cut-off by two sites, thus resulting in discordant negative *PGR* binary results (Figure 1C). In addition, *MKI67* dCt measurements were below the cut-off for two IHC-positive cases. In particular, case #2 was reported as discrepant mRNA negative by four sites, with the dCt of one site differing by only 0.1 from the cut-off, and case #10 was reported as discordant negative by one site, also with a difference from the cut-off of 0.1 (Figure 1E).

### 3.2. Re-Evaluation of Discrepant Cases

The two cases (case #2 and case #10) for which *PGR* and *MKI67* binary results were discrepant were re-evaluated regarding the histopathological features and the immunohistochemical expression of PgR and Ki-67.

**Figure 2:**
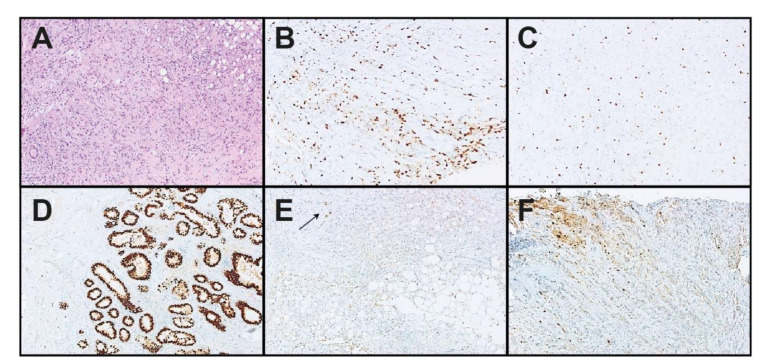
Histological review of one case reported as falsely negative for *PGR* and *MKI67* by two sites. Case #2 was a pleomorphic invasive lobular breast cancer. (**A**): Hematoxylin and eosin staining (×200). (**B**,**C**): Heterogeneous immunohistochemical (IHC) expression of Ki-67 (×200). (**D**): Internal positive control (non-neoplastic breast epithelium) in the progesterone receptor (PgR) IHC (×200). (**E**,**F**): Heterogeneous IHC expression of PgR (mostly negative, but focally positive with strong intensity) (×200).

Case #2 presented a pleomorphic invasive lobular breast cancer with heterogeneous low positive PgR (IHC staining < 10%), negative ER staining (0%), HER2-positive staining (IHC 3+), and heterogeneous Ki-67 expression with up to 30% positive staining on IHC (Figure 2). For this case, two EQA sites had reported discrepant *PGR* status as negative and four sites with negative *MKI67* status. IHC was repeated on deeper sections, showing lower PgR expression than in the initial PgR IHC. However, there was still focally very strong expression of PgR (IRS 3/12). The repeated Ki-67 IHC displayed less, but still, expression heterogeneity and confirmed high Ki-67. A repeated STRAT4 analysis of the deeper section delivered the same concordant results as the CC IHC and the first CC STRAT4 testing, with *PGR* and *MKI67* expression values clearly above the dCt cut-off. Yet, it should be noted that PgR IHC showed only low positivity near the cut-off with negative ER staining and Ki-67 a partly heterogeneous but high expression pattern. 

Case #10 was an IBC of no special subtype (NST) that displayed a partly inhomogeneous Ki-67 expression (up to 25–30%) in both the initial and the second (deeper cut) Ki-67 IHC of the CC. The initial STRAT4 testing in the CC had delivered an *MKI67* dCt of −3.7, regarded (given the cut-off of −4.0) as positive *MKI67* mRNA expression level, which is generally correlated with > 20% IHC Ki-67 staining, based on the Ki-67 IHC cutoffs used in prior STRAT4 validation studies [26,32]. However, one EQA site had reported *MKI67* as negative with a dCt value of −4.1, just below the cut-off (−4.0). In a second STRAT4 analysis of a deeper section performed by the CC, the reported *MKI67* dCt was −4.1, which is slightly below the cut-off. Hence, tumor heterogeneity should be considered in this case, since there were discrepant *MKI67* expression values in different adjacent sections of the same tumor tissue.

## 4. Discussion

Here, we presented the first Europe-wide retrospective Xpert^®^ Breast Cancer STRAT4 EQA study. In the study, mRNA-based assessments of *ESR1*, *PGR*, *ERBB2* and *MKI67* expression levels by a cartridge-based, multiplexed test were compared with the diagnostic gold standard, namely immunohistochemical expression of ER, PgR, HER2 (with additional ISH, if necessary), and Ki-67. The aim of our EQA study was to assess the quality and reproducibility of the STRAT4 test in various European institutes that had experience with the test as part of independent clinical studies.

STRAT4 testing was performed on adjacent tissue sections from ten pre-therapy breast core biopsies, containing IBC, by five European pathologic laboratories as well as the coordinating center pathology laboratory. Overall, each participating site passed the EQA criteria. While for *ESR1* and *ERBB2*, results were in perfect agreement with the IHC/ISH subtyping, sensitivity for *PGR* and *MKI67* were, respectively, 94% and 86%. Lower concordance of *PGR* and *MKI67* mRNA levels versus IHC using STRAT4 has been previously described, associated with mRNA/IHC expression levels in an interval close to the cut-offs [26]. In addition, a lower concordance rate for the markers *PGR* and *MKI67* has also been described for another mRNA-based test [33]. Our study thus suggests that, in addition to the binary test result, the absolute dCt value and its distance to the dCt cut-off should be considered when interpreting the STRAT4 test result for therapeutic stratification.

Lower concordance for *PGR* and *MKI67* was demonstrated in two cases that were reported as falsely negative for both markers by 2 and 4 sites, respectively. The initial IHC stains were reviewed and another IHC for both markers, as well as STRAT4 testing, were repeated on deeper sections. For one case (#2), both IHC and STRAT4 repeat testing confirmed the initial CC results. However, PgR IHC was only focally strongly expressed (low positive, < 10%). Therefore, this expression, very close to the IHC PgR positive cut-off of ≥1%, may have hampered *PGR* mRNA assessment. Repeat Ki-67 expression by IHC in the same case was high (30%), but with heterogeneous distribution, which may have caused false negative results. The second discrepant case displayed a partly inhomogeneous, but high (25–30%), Ki-67 protein expression on initial and repeat IHC testing. One participating site as well as the repeated STRAT4 test in the CC delivered a negative *MKI67* status on repeat IHC testing, which was, however, very close to the cut-off. Most other cases had clear-cut (far from cut-offs) positive or negative expression of ER, PgR, HER2, and Ki-67 in IHC/ISH. Hence, the study showed that mRNA-based testing was very reliable and reproducible in cases that had clear-cut IHC expression, but accuracy might decrease in cases in which expression was close to the predefined cut-offs. However, it should be noted that even for ER, PgR, HER2, and Ki-67 IHC/ISH, definitions of the respective optimal cut-offs are frequently under discussion. For instance, there is no standardized evaluation protocol nor one worldwide-accepted percent staining cut-off for Ki-67 to date [5,6,34], although efforts to harmonize Ki-67 assessment have been recently published [23].

Taken together, Xpert^®^ Breast Cancer STRAT4 mRNA testing for *ESR1*, *PGR*, *ERBB2*, and *MKI67* demonstrated good performance when compared to the diagnostic gold standard IHC/ISH. Hence, the mRNA test may be an efficient option for breast cancer subtyping, especially if a laboratory struggles with IHC/ISH evaluation due to pre-analytical, analytical, or post-analytical errors. Pre-analytical issues may be due to inadequate fixatives or time to and of fixation. Another issue may be the size of the tumor area and the low tumor cellularity, respectively, of breast core biopsies. For STRAT4 testing, mRNA automatically extracted from core biopsies is sufficient in most cases (unpublished data of the CC). Analytical errors of IHC/ISH may be caused by inadequate protocols or failure of reagents. Indeed, STRAT4 is a very easy to handle and robust test if implemented according to the manufacturer’s instructions. Concerning the reading of IHC/ISH by the pathologist, further problems may occur. First, the assessment may differ between readers causing inter-observer variability. Second, differences in staining procedures and antibody clones may lead to variable staining intensity/percentage. Furthermore, scoring methods, as mentioned above, may differ.

Our EQA study included only ten tumor samples, as is often the case in EQA studies [35,36]. It would be interesting to assess the reliability, i.e., the performance relative to reference tests, as well as the predictive and prognostic value of the assay in extensive clinical cohorts. This is currently being performed in a single-center study. Moreover, lacking variability of ER expression and missing an HER2 IHC2+/ISH amplified case are limitations of this study but was due to the fact that we concentrated on different Ki-67 expression levels.

## 5. Conclusions

Our EQA study shows that a cartridge-based mRNA assay might offer a reproducible, standardized and time- and tissue-sparing alternative for the evaluation of *ESR1* (ER), *PGR* (PgR), *ERBB2* (HER2), and *MKI67* (Ki-67) in IBC. Particularly, it may be an alternative test for consideration if the establishment and implementation of IHC/ISH is hampered due to (pre-/post-)analytical or economic reasons. However, tumor heterogeneity and expression patterns by IHC that are close to the defined test cut-offs may deliver false negative or false positive results for cases with borderline reference test results. Hence, reliability, reproducibility, and the assay’s prognostic and predictive value regarding pathological complete response should be investigated in large clinical breast cancer cohorts. Currently, we are analyzing this issue in a single-center study.

## Figures and Tables

**Table 1:** IHC/ISH subtyping of the EQA study cohort. Positivity of either ER and/or PgR are summarized as hormone receptor-positive (HR+). *CEN17*: centromere of the corresponding chromosome 17; n.p.: not performed.

Sample Information		ER IHC			PgR IHC			HER2 IHC/CISH			Ki-67 IHC	
EQA Sample ID	Subtype by IHC	ER IHC Status	ER IHC Percentage	ER IHC IRS	PgR IHC Status	PgR IHC Percentage	PgR IHC IRS	HER2 IHC/CISH Status	HER2 Dako Score (IHC)	*HER2* CISH	Ki-67 IHC Status	Ki-67 IHC Percentage (%)
1	TNBC	negative	0	0	negative	0	0	negative	1	n.p.	positive	30
2	HER2+	negative	0	0	positive (low)	<10	3	positive	3	n.p.	positive	30
3	HR+, Ki-67 low	positive	95	12	positive (low)	<10	1	negative	1	n.p.	negative	5
4	HR+, Ki-67 low	positive	90	12	positive	90	12	negative	1	n.p.	negative	15
5	HR+, Ki-67 high	positive	95	12	positive (low)	<10	3	negative	0	n.p.	positive	50
6	TNBC	negative	0	0	negative	0	0	negative	1	n.p.	positive	80
7	HER2+	negative	0	0	negative	0	0	positive	3	n.p.	positive	50
8	HR+, Ki-67 low	positive	80	12	positive	70	6	negative	0	n.p.	negative	15
9	HR+, Ki-67 high	positive	95	12	positive	60	9	negative	2	Negative (*HER2/* *CEN17* ratio = 0.87)	positive	30
10	HR+, Ki-67 high	positive	95	12	positive	100	12	negative	1	n.p.	positive	30

**Table 2:** Comparison of IHC/ISH status with the STRAT4 binary results. The table shows the average sensitivity, specificity, precision (PPV), and accuracy across all five participating sites (in parenthesis the 95% confidence interval). PPV: positive predictive value.

Performance Index	ER	PgR	HER2	Ki-67
**Sensitivity**	1 (1–1)	0.94 (0.87–1)	1 (1–1)	0.86 (0.76–0.95)
**Specificity**	1 (1–1)	1 (1–1)	1 (1–1)	1 (1–1)
**Precision (PPV)**	1 (1–1)	1 (1–1)	1 (1–1)	1 (1–1)
**Accuracy**	1 (1–1)	0.96 (0.91–1)	1 (1–1)	0.9 (0.83–0.97)

## Data Availability

On request, the data presented in this study are available as summary Excel format from the corresponding authors.
